# Isolation of HDL by sequential flotation ultracentrifugation followed by size exclusion chromatography reveals size-based enrichment of HDL-associated proteins

**DOI:** 10.1038/s41598-021-95451-3

**Published:** 2021-08-09

**Authors:** Jack Jingyuan Zheng, Joanne K. Agus, Brian V. Hong, Xinyu Tang, Christopher H. Rhodes, Hannah E. Houts, Chenghao Zhu, Jea Woo Kang, Maurice Wong, Yixuan Xie, Carlito B. Lebrilla, Emily Mallick, Kenneth W. Witwer, Angela M. Zivkovic

**Affiliations:** 1 Department of Nutrition, University of California, Davis, Davis, CA USA; 2 Department of Chemistry, University of California, Davis, Davis, CA USA; 3 Department of Molecular and Comparative Pathobiology, Johns Hopkins University School of Medicine, Baltimore, MD USA

**Keywords:** Analytical biochemistry, Isolation, separation and purification, Mass spectrometry, Proteomic analysis, Structure determination

## Abstract

High-density lipoprotein (HDL) particles have multiple beneficial and cardioprotective roles, yet our understanding of their full structural and functional repertoire is limited due to challenges in separating HDL particles from contaminating plasma proteins and other lipid-carrying particles that overlap HDL in size and/or density. Here we describe a method for isolating HDL particles using a combination of sequential flotation density ultracentrifugation and fast protein liquid chromatography with a size exclusion column. Purity was visualized by polyacrylamide gel electrophoresis and verified by proteomics, while size and structural integrity were confirmed by transmission electron microscopy. This HDL isolation method can be used to isolate a high yield of purified HDL from a low starting plasma volume for functional analyses. This method also enables investigators to select their specific HDL fraction of interest: from the least inclusive but highest purity HDL fraction eluting in the middle of the HDL peak, to pooling all of the fractions to capture the breadth of HDL particles in the original plasma sample. We show that certain proteins such as lecithin cholesterol acyltransferase (LCAT), phospholipid transfer protein (PLTP), and clusterin (CLUS) are enriched in large HDL particles whereas proteins such as alpha-2HS-glycoprotein (A2HSG), alpha-1 antitrypsin (A1AT), and vitamin D binding protein (VDBP) are enriched or found exclusively in small HDL particles.

## Introduction

**Figure 1:**
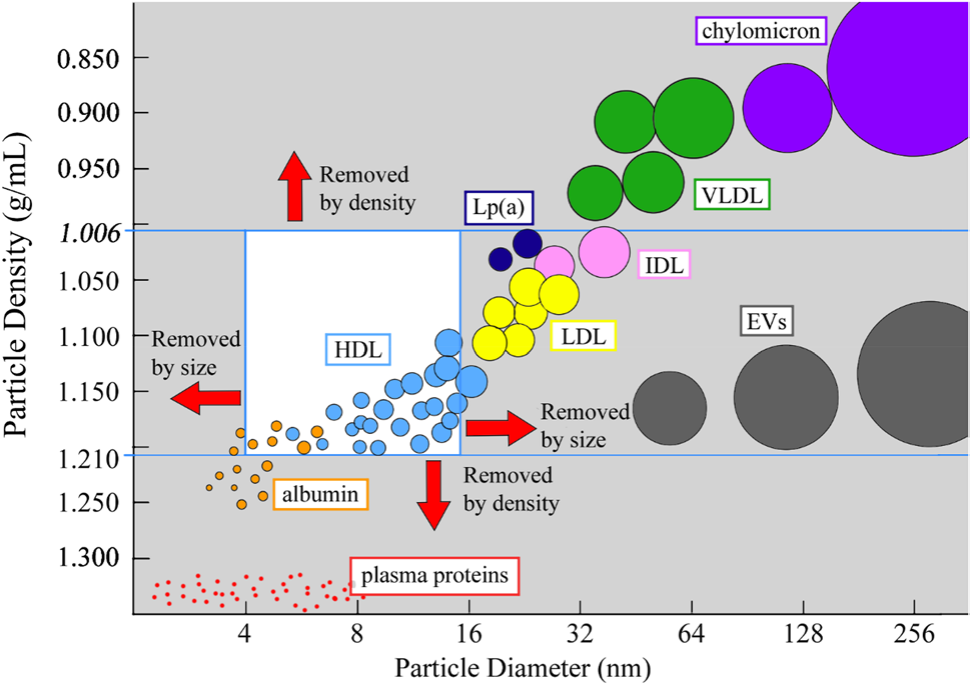
Schematic of the size and density range of lipoproteins and low-density plasma components. The white box indicates the density and size of high density lipoprotein (HDL) particles captured by this HDL isolation method, as well as the particle diameter (nm) and particle density (g/mL) of the other lipoprotein particles—chylomicrons, very low density lipoproteins (VLDL), intermediate density lipoproteins (IDL), low density lipoproteins (LDL), lipoprotein (a) (Lp(a)), and extracellular vesicles (EVs) present in plasma that are simultaneously isolated as part of this method.

High Density Lipoprotein (HDL) particles are the primary cholesterol scavenging vehicles most known for their role as mediators of cardiovascular health and their involvement in attenuating the progression and pathogenesis of cardiovascular disease (CVD)^1^. Epidemiological studies have shown a negative correlation between serum HDL-cholesterol levels and the risk of coronary heart disease^2^. Despite HDL’s well-known clinical significance, basic research on HDL function and composition has produced many confusing and inconsistent results. This is in part due to the difficulty of isolating functional HDL from plasma that represents the diversity of HDL particles, preserves particle integrity, and is uncontaminated by other plasma components^3^. There is a renewed interest in lipoprotein particles due to the recent discovery that they are carriers of extracellular RNA^4^. The extracellular RNA carriers in plasma (i.e. lipoprotein particles and extracellular vesicles (EVs)) span the range from as small as 7 nm in radius to as large as 1 micron in radius, and from a density of 1.21 g/mL to close to plasma density at 1.006 g/mL (Fig. 1). Classically, HDL is isolated by ultracentrifugation (UC) based on the finding that HDL particles float in the density range of 1.063–1.210 g/mL, which allows their separation from less dense lipoprotein particles such as chylomicrons, very-low density lipoprotein (VLDL), intermediate-density lipoprotein (IDL), and low-density lipoprotein (LDL), and denser serum proteins^5^. However, if HDL particles are separated by density alone, they overlap the density range of some LDL (1.019–1.063 g/mL, Fig. 1) and if they are isolated by size alone, they overlap the size range of many plasma proteins (e.g. ferritin at about 12 nm radius). A plethora of additional isolation strategies have been reported including size exclusion chromatography (SEC)^6,7^, immunoaffinity precipitation^8–10^, chemical precipitation^11,12^, asymmetrical flow field flow fractionation (AF4)^13^, and approaches combining these techniques^4,14,15^.

Careful selection of HDL isolation method is critical as different isolation methods can produce starkly different yields and experimental results. For example, depending on the isolation method, the HDL proteome has been reported to contain as many as > 100 proteins^3^, or as few as a dozen^15^. Given that each HDL isolation method has its own advantages and disadvantages, and that the results of a study will vary based on the method, there are a number of variables to consider when choosing an isolation method. For example, while UC-based HDL isolation has the advantage of being scalable to yield high quantities of HDL, prolonged UC duration can damage particle structure and result in the loss of certain attached proteins^16,17^. On the other hand, while direct SEC-based separation from plasma is a gentler extraction procedure, without further clean-up there can be extensive contamination with LDL and serum proteins^6^. Immunoaffinity-based HDL isolation using antibodies to HDL proteins can offer a high degree of both sensitivity and specificity but produces low yields and is expensive. One of the newest and most promising methods for HDL isolation is asymmetric-flow field-flow fractionation (AF4), which has the advantages of gentle extraction and excellent reproducibility^13^. However, the method requires advanced instrumentation and technical expertise that is not yet widely available. Many of these techniques also lack adjustability. Depending on the research question and logistical limitations of the particular research project, the parameters that need to be optimized by the HDL isolation method may differ because often maximizing for certain parameters necessitates compromising on other parameters. For example, maximizing for purity typically means loss of particle diversity, whereas maximizing yield often means loss of purity.

**Figure 2:**
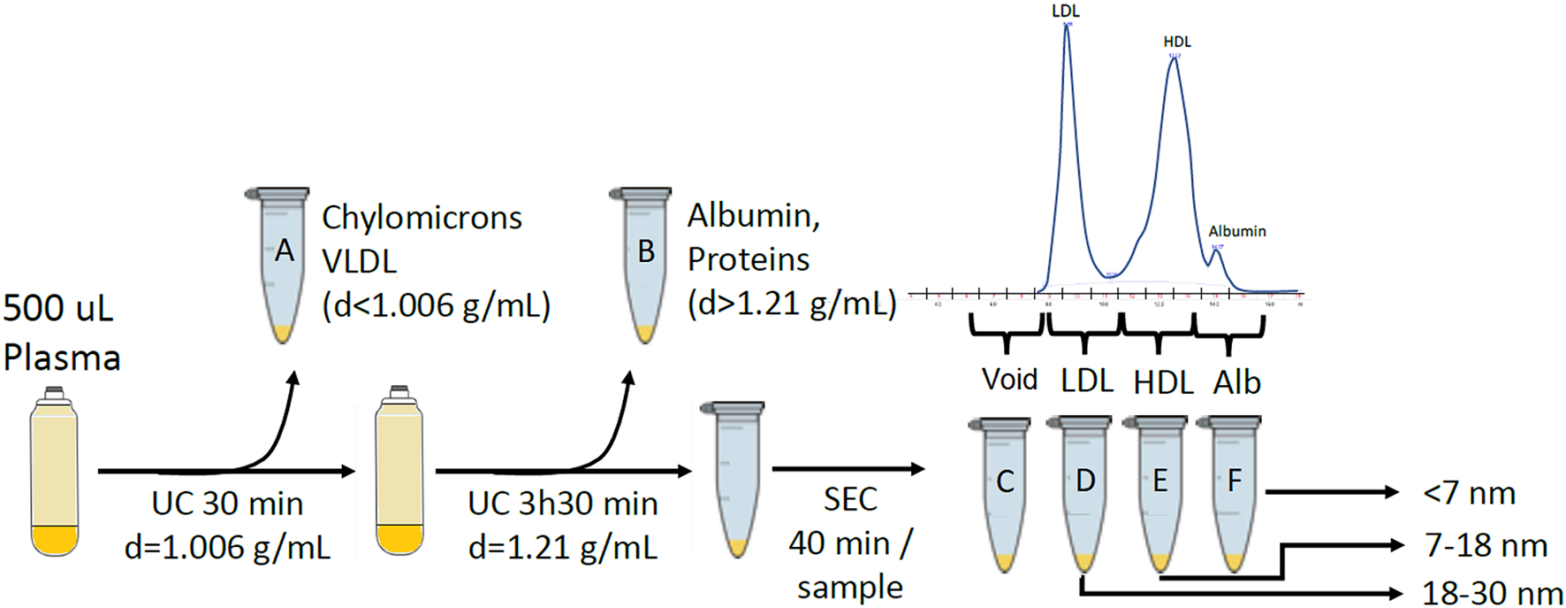
Visual abstract of the isolation method. A starting plasma volume of 500uL is first subjected to sequential flotation ultracentrifugation (UC) at a density of d = 1.006 g/mL to float off the chylomicrons and very low density lipoproteins (VLDL) (**A**: particles with density d < 1.006 g/mL), followed by a second UC at a density of d = 1.21 g/mL to precipitate out the albumin and other plasma proteins (**B**: proteins with density d > 1.21 g/mL). The resulting supernatant of density in the range of 1.006–1.21 g/mL is then separated by size exclusion chromatography (SEC) to yield intermediate density lipoproteins (IDL)) (**C**: size range of 30–200 nm in diameter), low density lipoproteins (LDL) (**D**: size range of 18–30 nm in diameter), high density lipoproteins (HDL) (**E**: size range of 7–18 nm in diameter), and albumin (**F**: size < 7 nm in diameter).

Here we report an HDL isolation method, suitable for the purpose of isolating HDL from human plasma samples for multiple simultaneous structural, compositional, and functional assays. The method combines sequential flotation UC followed by SEC, based on a previously published method using density gradient UC followed by SEC^15^. The method described here separates the HDL first by density from triglyceride-rich particles and serum proteins, then isolates the HDL by size from LDL and albumin (Fig. 2). The method yields highly purified HDL fractions verified by polyacrylamide gel electrophoresis (PAGE), transmission electron microscopy (TEM), and proteomics. The advantages of this method include: (1) ability to fine tune the HDL fraction(s) of interest, from the least inclusive but purest fraction to pooling multiple fractions in order to capture particle heterogeneity; (2) preservation of particle integrity due to minimal centrifugation duration (a total of 4 h) and lack of introduction of chemical or other modifying agents, making it amenable for in vitro functional assays and particle size and structural analysis; (3) a low starting plasma volume requirement (500 uL or less but with lower HDL yield), making it suitable for clinical studies with limited sample availability such as studies using samples from biorepositories or previously conducted cohort studies; (4) high yield—a concentrated 100 uL aliquot of HDL fractions at a concentration of on average 4 mg/mL protein (400 µg total protein) which can be divided into several aliquots for multiple simultaneous compositional and functional analyses; (5) high throughput—separation of 8 samples within 12 h; and (6) high purity—a demonstrated minimal contamination from serum proteins and other lipoprotein particles including LDL. In addition, this method simultaneously allows for the isolation of chylomicrons/VLDL, IDL, LDL, serum proteins, and lipidated albumin from the same starting plasma, which means that precious samples can be maximally mined.

## Methods

The objective of this method is to isolate HDL particles via a 2-step process including a sequential flotation UC step to remove triglyceride-rich particles and plasma proteins by density, followed by SEC using an FPLC system to separate the lipoproteins by size. The aim of the UC step is to isolate plasma particles in the density range of 1.006–1.210 g/mL, which includes IDL, LDL, HDL, and albumin, but excludes chylomicrons, VLDL, and plasma proteins. The aim of the SEC step is to isolate the HDL particles from both the larger particles (i.e. LDL) and the smaller particles (i.e. albumin) using a SEC column. Figure 1 illustrates the size (radius in nm) and density ranges (in g/mL) of plasma proteins and lipoproteins, and indicates the specific density and size range that this UC-SEC method achieves for HDL isolation and purification.

### Biological sample collection

Pooled human plasma samples from a previously conducted trial were used for the method development in this study. The trial was a cross-over, double blinded investigation of the effects of 4 weeks supplementation with a dietary fiber supplement vs. placebo. Only the baseline (i.e. pre-intervention) plasma samples were used to generate the plasma pool. The subjects were young healthy individuals meeting the following inclusion and exclusion criteria: between 18 and 45 years of age, body mass index ranging from 23.0 to 32.0 kg/m^2^, non-smokers, not pregnant or breastfeeding, have not started or changed birth control in the last 6 months, have not taken antibiotics in the last 6 months, not taking prescription medications including statins and blood pressure medications, no history of chronic disease including diabetes, thyroid disease, metabolic syndrome, irritable bowel syndrome, Crohn’s disease, ulcerative colitis, any inflammatory disease, hypertension, cancer, or cardiovascular events, do not have anemia or difficulty with blood draws, typically consume one or less alcoholic drinks per day and participate in binge drinking (more than three alcoholic drinks in one episode) at most one day per month, agree to abstain from alcohol for two days before their blood draw, had not experienced any immunosuppression at the time of enrollment, and had not experienced cold or flu two weeks prior to the blood draw. The subject characteristics including their lipid profiles are summarized in Supplemental Table S1. The study was approved by the Institutional Review Board of UC Davis. The study followed all approved guidelines for the protection of subjects and is registered with ClinicalTrials.gov with NCT #03785860. Participants who qualified for the study were enrolled in the study and provided written informed consent. After consent subjects were scheduled for a visit to the Ragle Human Nutrition Research Center on the UC Davis campus. A fasting blood draw was performed from the antecubital vein into ethylenediaminetetraacetic acid (EDTA) evacuated tubes, immediately centrifuged (1500×*g*, 10 min, 4 °C), portioned into aliquots, and stored at − 80 °C. Baseline plasma from the first ten subjects recruited into this study were used to generate a plasma pool. Frozen plasma samples were thawed on ice for 2 h, combined in a sterile 50 mL falcon tube (Fisher Scientific), stir-mixed, separated into 500 µL aliquots, and stored at – 80 °C until analysis.

Additional samples were used to demonstrate the repeatability of the method. These samples are pooled plasma from the Extracellular RNA Communication Consortium (ERCC2) project, which is an NIH Common Fund project aimed at the development of isolation and characterization methods for extracellular RNA carriers (https://commonfund.nih.gov/exrna), including extracellular vesicles and lipoproteins. Plasma was obtained from healthy adult participants in San Diego, CA under an approved IRB from the University of California San Diego. Frozen, de-identified plasma from 25 individuals (men and women) was thawed on ice for 2 h, combined in a sterile 50 mL falcon tube (Fisher Scientific), stir-mixed, separated into 500 µL aliquots, and stored at − 80 °C until analysis.

### Ultracentrifugation for separation of chylomicron/VLDL and plasma proteins

Density solutions were prepared at 1.0060 g/mL (1.095% potassium bromide (KBr) w/w in HPLC grade water), 1.2100 g/mL (9.439% KBr w/w in HPLC grade water), and 1.3400 g/mL (59.112% KBr w/w in HPLC grade water), (± 0.0005 g/mL) using anhydrous KBr (Sigma-Aldrich, MO, USA). The density of prepared KBr solutions was measured at ambient temperature (22.8 °C) with a Densito 30PX Densitometer (Mettler Toledo, OH, USA) in triplicate. The density solutions were filtering through a 0.22 µm filter into a sterile bottle. Plasma samples in 500 µL aliquots were thawed at 4 °C for 16 h followed by pipet-mixing. The 500 µL homogeneous plasma was underlaid beneath 4.1 mL 1.0060 g/mL KBr density solution in a 4.7 mL OptiSeal tube (Beckmann-Coulter, IN, USA). Extra 1.0060 g/mL KBr solution was added to the top of the tube until the surface meniscus reached the neckline of the OptiSeal tube. The filled OptiSeal tube was then sealed with a disposable plug that is complementary with the OptiSeal tube, and securely locked by a spacer (Beckmann-Coulter). The layered solution was then loaded onto a fixed angle rotor (TLA-110 Fixed-Angle Rotor, k factor = 13, Beckman-Coulter) and centrifuged at 110,000 RPM (657,272×*g*) in a Beckman Optima MAX-TL ultracentrifuge (Beckmann-Coulter) at 14 °C for 30 min. After centrifugation, the top 1 mL layer was removed with the tip of a pipette drawing solution from the surface while rotating the OptiSeal tube, then the tube was cut below the neck with tubing cutter to expose the remaining solution for accessibility, and 3 additional milliliters were removed to ensure floating particles were removed. The removed 4 mL layer containing chylomicron and VLDL could be saved for future use. To isolate the plasma proteins, the remaining 700 µL fraction containing HDL, LDL, albumin, and plasma proteins was then fully mixed with 1.1 mL 1.340 g/mL KBr density solution by pipetting. The 1.9 mL homogeneous solution was then underlaid carefully beneath 2.8 mL 1.210 g/mL KBr density solution in an OptiSeal tube. Extra 1.210 g/mL KBr solution was added from the top to fill the solution until the surface meniscus reached the neckline of the OptiSeal tube. The OptiSeal tube was sealed and locked as described above, and steadily loaded onto the same rotor, and centrifuged using the same parameters for 3.5 h.

In order to assess the effectiveness of the SEC purification step of this method, an additional UC-only isolation, using the same equipment and supplies as described above, was carried out on a subset of 2 individuals at 2 time points (a total of 4 samples). The samples were first adjusted to the LDL density cutoff of 1.063 g/mL with KBr solution (d = 1.340 g/mL) and the adjusted plasma was overlaid with KBr solution (d = 1.063), followed by ultracentrifugation at 110,000 rpm for 3 h 10 min to isolate the combined chylomicron, VLDL and LDL fraction, and removal by aspiration of the supernatant. The remaining fraction was then adjusted to 1.210 g/mL with KBr solution (d = 1.34 g/mL) and overlaid with KBr solution (d = 1.210 g/mL), followed by a second ultracentrifugation at 110,000 rpm for 3 h 20 min. The HDL fraction (1.063–1.210 g/mL) was then carefully collected by pipette from the top of the tube.

### Dialysis and size-exclusion chromatography

After the second UC step, the top 2 mL of the solution was collected carefully by pipette while slowly turning the tube and mixed with 2 mL of molecular grade 1X Phosphate Buffered Saline (1XPBS, [NaCl]: 137 mM; [KCl]: 2.7; [Na_2_HPO_4_]: 10 mM; [KH_2_PO_4_]: 1.8 mM) (Sigma-Aldrich). The mixture was filtered through an Amicon Ultra-4 50 kDa centrifugal filter (Millipore) at 4500 RPM (1100×*g*) for 8 min using a Sorvall Legend XF centrifuge (Thermo Fischer Scientific, MA, USA). The HDL fraction was reconstituted to 250 µL with millipure water and then transferred to an amber glass vial for FPLC analysis.

Samples were separated with a single Superdex 200 Increase 10/300 GL agarose-crosslinked column (GE Healthcare) on an AKTA P-920 FPLC (Amersham Biosciences). Two hundred microliters of each sample were injected into the system under isocratic elution (1.0 mL/min, 1XPBS.), the first 6.5 mL were discarded, then a 1.5 mL-fraction (including a 0.5 mL void volume) was collected (F0), followed by collection of 6 1-mL fractions (F1-F6) and 1 0.5-mL fraction (F7, excluding a 0.5 mL void volume). The elution was measured continuously at 280 nm UV absorbance, and temperature, conductivity and pH were recorded. UV absorbance was used as a proxy for particle concentration to visually confirm fractionation of the LDL, HDL, and albumin peaks. The eight collected fractions correspond to the pre-LDL fraction (F0), the LDL peak (F1–F2), the HDL peak (F3–F6) and the albumin peak (F7). Each fraction was dialyzed in an Amicon Ultra-4 50 kDa centrifugal filter (Millipore) at 4500 RPM (1100×*g*) for 8 min.

In order to evaluate the reproducibility of our method across different subjects with different lipid profiles, individual plasma samples from three healthy participants whose plasma was used to generate the plasma pool was selected, and their HDL isolated using a fractionation plan manually adjusted to the elution volume at which the troughs between the LDL and HDL peaks, and between the HDL and albumin peaks were present. This was a different fractionation plan compared to the 1-mL fractionation described above, and it was set this way to maximize HDL yield and minimize the inclusion of albumin and other small molecules. For these three individual subjects the LDL fractions (F1 and F2) were manually set at between 8.85 and 9.78 mL, the HDL fractions (F3 and F4) were manually set at between 9.78 and 13.26 mL, and the albumin fraction (F5) was manually set at between 13.26 and 15.00 mL (Supplemental Fig. S2).

In order to evaluate the repeatability of the method over time, we used plasma samples pooled from the ERCC2 project as described above to serve as a quality control standard. This plasma quality control is isolated every 10 samples when running a large batch of samples, representing chromatograms of the same sample over several weeks of individual runs. The elution volume for LDL, HDL, and albumin was determined at the max 280 nm UV absorbance for each peak.

### Protein concentration measurement

Fraction F3-F6 were selected as HDL fractions, and the total protein concentration in these fractions were used to assess the yield of HDL by our isolation method. Protein concentration was determined using a commercially available microBCA Protein Assay kit (Thermo Scientific). Briefly, a standard curve of bovine serum albumin (BSA) with the concentration of 200, 40, 20, 10, 5, 2.5, 1, 0.5, and 0 µg/mL was prepared in duplicate. One-hundred and fifty microliters of BSA standard or HDL samples were loaded on a clear-bottom 96-well microplate (Greiner Bio-One), and mixed with 150 µL Working Reagent (provided) for a 2-h incubation at 37 °C. After the incubation, the absorbance of the solution was measured at 562 nm and recorded by a SpectraMax M5 microplate reader (Molecular Devices). The absorbance data for the BSA standard were plotted against the BSA concentration to obtain a standard curve. The molar extinction coefficient was determined by the “Trendline” function in Microsoft Excel. The protein concentration in HDL fractions F3-F6 were then calculated by dividing the adjusted absorbance by the molar extinction coefficient of the BSA standards according to Beer’s Law.

### Verification of purity of the isolated HDL fractions

Detailed protein composition in each SEC fraction was identified by proteomics, which was chosen as an unbiased evaluation of the protein composition of isolated HDL fractions. The isolated HDL fractions were reconstituted with 50 μL of 50 mM ammonium bicarbonate and denatured with the additional 2 uL of 550 µM dithiothreitol. It was then added to the samples and incubated for 50 min at 55 °C. The free thiol groups were then alkylated with 4 μL of 450 mM iodoacetamide for 25 min at room temperature in the dark. The mixture was digested by adding 2 μg trypsin in 200 µL of 50 mM ammonium bicarbonate and incubated for 18 h at 37 °C. The digested peptides were purified by solid-phase extraction with Supelclean C18 cartridges containing 500 mg sorbent materials (Sigma-Aldrich), and the samples were dried in vacuo using a miVac sample concentrator (SP Scientific, PA, USA). Tryptic digested samples were reconstituted with 20 μL of water and directly characterized using UltiMate WPS-3000RS nanoLC 980 system coupled to the Nanospray Flex ion source of an Orbitrap Fusion Lumos Tribrid Mass Spectrometer (MS) system (Thermo Fisher Scientific). One microliter of the sample was injected, and the analytes were separated on an Acclaim PepMap 100 C18 LC Column (3 μm, 0.075 mm × 150 mm). A binary gradient was applied using 0.1% (v/v) formic acid in (A) water and (B) 80% acetonitrile: 0–90 min, 4–47% (B); 90–100 min, 47–70% (B); 100–101 min, 70–100% (B); 116–117 min, 100–4% (B). The instrument was run in data-dependent mode with 1.8 kV spray voltage, 275 °C ion transfer capillary temperature, and the acquisition was performed with the full MS scanned from 375 to 2000 in positive ionization mode. Higher-energy C-trap dissociation at 35% was applied to obtain tandem MS/MS spectra with *m/z* values starting from 120.

The proteins were identified using Byonic software (Protein Metrics, CA, USA), and the *Homo sapiens* (Human) protein database from UniProt (UP000005640) was used for the protein database file. Carbamidomethyl modification at cysteine residues (set as fixed) and oxidation at methionine (set as variable) were assigned as the modification. The resulting files were further loaded to Byologic software (Protein Metrics) for protein quantification. Proteins detected by a minimum of two unique peptides were used in the analysis. Known typical contaminants of the proteomics process including keratin (K1C14, K2C6B, K2C1, K1C10, K2C5, K1C9, K22E, K2C1B) trypsin (TRY1, TRY3, and TRY6), and protein shroom 3 (SHRM3) were manually excluded from analysis. For each identified protein, the peptide with the highest signal and without any post-translational modifications was chosen as the quantitation peptide. The intensity from the extracted ion chromatogram (XIC) was used for protein quantification.

For SDS-PAGE, samples from each SEC fraction were first adjusted to about 0.4 mg/mL concentration with water. Fifteen microliters of each SEC fraction (F0–F7) were mixed with 15 µL of Lane Marker Reducing Sample Buffer (Thermo Fisher Scientific) and heated on a heat block (100 °C) for five minutes. Then, the unfolded and reduced fractions were loaded into a 4–20% Mini-PROTEAN Precast polyacrylamide gel (BioRad), with Page Ruler Prestained Protein Ladder (Thermo Fisher Scientific). The gels were run at 120 V for about 60 min, then removed from the plastic casing, stained with InstantBlue (Sigma-Aldrich) protein stain for 45 min, and ChemiDoc MP imaging instrument (Bio-Rad) The image of the gel was then cropped using the Image Lab software.

For native-condition PAGE, the isolated fractions were first diluted into 0.4 mg/mL with water. The solutions were then mixed with native sample buffer (BioRad) in 1:1 (v/v) ratio. Then, 20 mL containing about 5 µg protein from each fraction was loaded into a 4–20% Mini-PROTEAN Precast polyacrylamide gel (BioRad). The sample in each well was run at 25 A for 1 h with Tris–glycine buffer without SDS (Genessee Scientific, San Diego, CA).

To quantify ApoA-I Western blot was performed. A nitrocellulose membrane (Biorad) was first activated with 100% methanol for 5 s on each surface. The activated membrane was then equilibrated in the Western blot transfer buffer (20% methanol, 20% Trans-Blot transfer buffer 5x (Biorad), 60% water (v/v/v). Two transfer stacks were also equilibrated in the transfer buffer for 5 min until fully soaked. The Western blot protein transfer sandwich was then assembled in the order of transfer stack, membrane, poly-acrylamide gel, transfer stack from the anode to the cathode in a Trans-Blot Turbo Transfer System case (Biorad). Protein transfer was run by selecting the “mix MW” mode, which provides 1.3 A for 7 min duration. The membrane was taken out and washed with TBST buffer (10% tris-buffer saline 10x (Biorad), 0.1% Tween (Biorad), 89.9% water, v/v/v) for 5 min each time, 3 times. The membrane was then incubated in 5% skim milk in TBST buffer for 1 h, followed by 3 5-min washes. Ten microliters of primary antibody solution (0.1% mouse anti-human ApoA-I antibody (Invitrogen, Waltham, MA), 5% bovine serum albumin (Biorad), 94.9% TBST, v/w/v) was added to the membrane for primary antibody incubation at 4 °C for 24 h. After 24 h of primary antibody incubation, the membrane was washed 3 times with TBST buffer, and incubated in 10 mL secondary antibody solution (0.01% goat anti-mouse HRP-linked antibody (Invitrogen), 5% skim milk, 94.95% TBST, v/w/v) for 1 h at room temperature. The membrane was washed 3 times with plenty of TBST for 5 min each time, and then 6 mL of HRP substrate (50% Clarity Western Peroxide Reagent, 50% Clarity Western Luminol/Enhancer Reagent) was added on the surface of the membrane. The reaction was captured using ChemiDoc MP imaging instrument (Bio-Rad) under “Chemiluminescent” mode.

### Verification of HDL Morphology and Cholesterol Efflux Capability

TEM was performed to visualize the particle morphological characteristics and size in each fraction. Carbon-coated grids (TedPella Inc., CA, USA) were glow-discharged at 30 mA for 30 s. Five microliters of fractions F1-F7 from the FPLC were loaded onto the carbon-coated side of the grid, allowed to sediment on the grid for 5 min, and blotted with filter paper to remove excess sample. The grid was then washed and stained with 5 µL 2% (v/v) uranyl acetate solution and blotted five times and left to air-dry at room temperature for 2 min. The dried grid was transferred onto the specimen cartridge on the specimen holder of the electron microscope (JEOL USA 1230 Transmission Electron Microscope, JEOL USA Inc., MA, USA) and inserted into the electron microscope according to the manufacturer's instructions. The sample is inserted into to the specimen chamber of the EM after vacuum is maintained. Each specimen was first viewed using the binoculars on the electron microscope under the condition of high tension = 120 kv and Magnitude = 40 K. When a section of the specimen contained a considerable amount of particles (≥ 20 particles in view), an image was taken with exposure time = 500 ms. Five images from different grid regions were taken for each specimen.

The cholesterol efflux capability of the isolated HDL was compared to that of a traditionally prepared ApoB-depleted plasma. Cholesterol efflux ability of HDL is known as the best functional assay to examine the atheroprotective capacity of HDL^18^. The experimental procedure was followed according to the protocol of a cholesterol efflux assay kit (Abcam, United Kingdom) with some modifications. Briefly, THP-1 human macrophage cells were plated into a microplate at a concentration of 10^5^ cells/well. The cells were then activated using 100 µL RPMI with 10% fetal bovine serum and 40 nM (64 ng/mL) phorbol-12-myristate 13 acetate (PMA) and incubated for 24 h at 37 °C and 5% CO_2_. After incubation, cells were washed with 1X PBS, and incubated with serum-free RPMI and labeling reagent (3:1) provided by the commercial kit for 4 h. After incubation, the labeling reagent was discarded, the wells were washed with 1X PBS, and serum-free RPMI with isolated HDL at 0.1 mg/mL of total protein concentration, measured by microBCA (Thermo Science) or ApoB-depleted plasma were loaded into the designated well. The plate was then incubated for 4 h. After incubation, the supernatant portion in each well was transferred to a new plate. The remaining cells in the wells were lysed with mammalian protein extraction reagent (Thermo Scientific, USA). Fluorescence emission of both the supernatant and the cell lysis portion were measured at 515 nm after 482 nm excitation. The cholesterol efflux to the cholesterol acceptor (HDL or ApoB-depleted plasma) was calculated by dividing the supernatant fluorescence emission by the sum of the fluorescence emission of the supernatant and cell lysis fractions.

## Results

**Figure 3:**
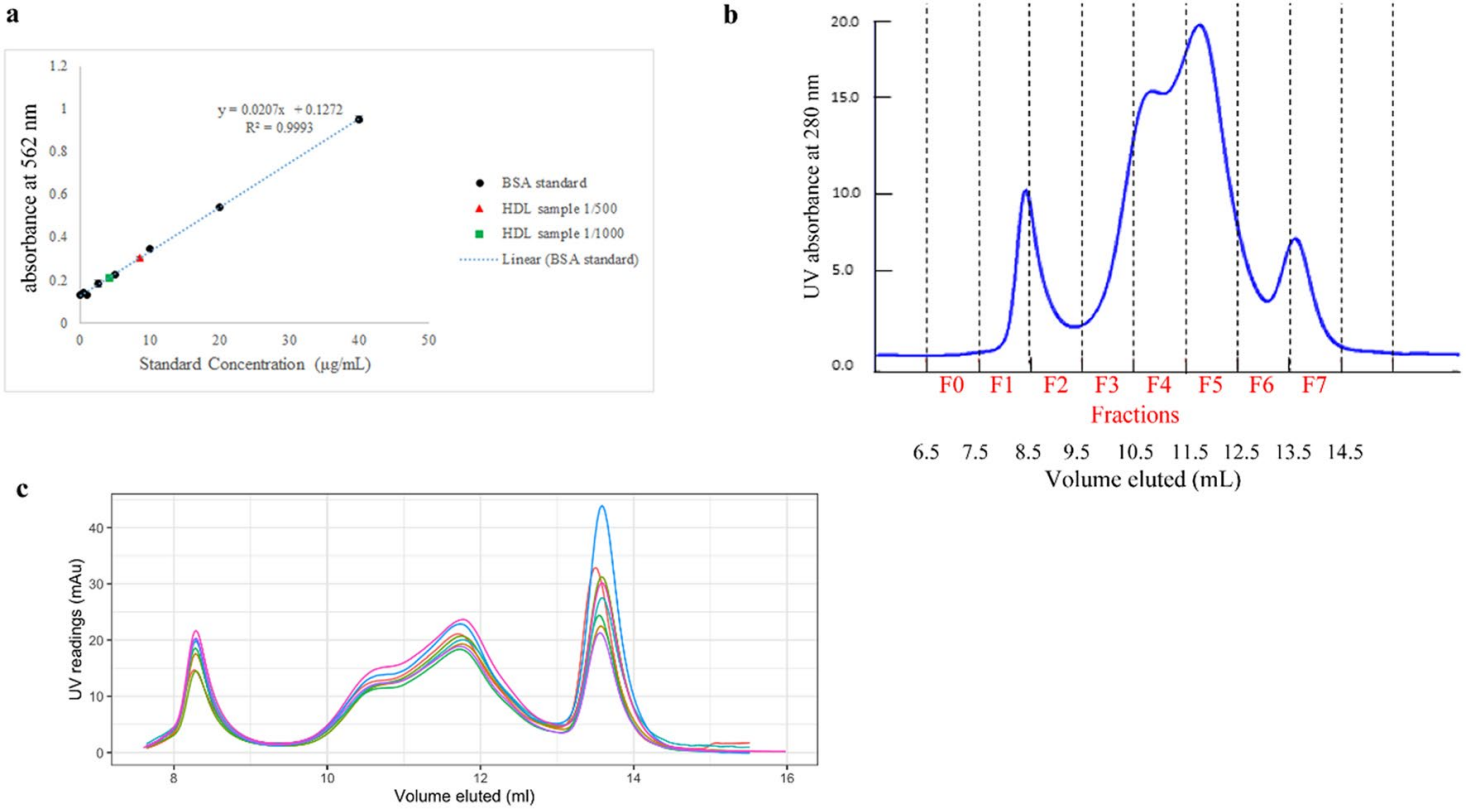
Overall HDL total protein concentration of size exclusion chromatography (SEC) fractions with SEC chromatogram. (**a**) The total protein concentration of diluted HDL samples (500- and 1000-times) calculated from a fitted bovine serum albumin standard curve. The concentration for the 500- and 1000-times diluted HDL samples were calculated to be 8.57 ± 0.31 µg/mL and 4.08 ± 0.17 µg/mL, respectively. (**b**) SEC chromatogram of pooled plasma sample following sequential flotation ultracentrifugation, with fractionation set to collect 1-mL fractions starting at 6.5 mL. Fractions F1 and 2, correspond to the low density lipoprotein (LDL) particle size range; Fractions F3-6, correspond to the high density lipoprotein (HDL) particle size range, with fraction F6 containing a portion of the albumin peak; Fraction F7 corresponds to the albumin peak. (**c**) A chromatogram showing 8 repeated SEC on plasma aliquots from a plasma pool on several different days to demonstrate the technical reproducibility of the separation procedure. The mean ± standard deviation peak elution volume for the LDL peak, HDL peak, and albumin peak are 8.28 ± 0.007, 11.7 ± 0.027, and 13.6 ± 0.029 mL, respectively. The % coefficient of variation of these are 0.09%, 0.23%, and 0.21%, respectively.

The HDL isolation method described here yields a set of highly purified HDL fractions that can either be combined to yield a single pool of total HDL or be used as distinct size-based subfractions of HDL. The total protein concentration in the 100 µL concentrated combined HDL fraction (F3-F6) was measured by microBCA protein assay to be 4.2 ± 0.1 mg/mL (Fig. 3a) (a total of about 400 µg protein).

As shown in the SEC chromatogram (Fig. 3b), the HDL particles eluted as a distinct double peak (F3–F6) with a smaller peak in fraction F4 and the apex of the peak in fraction F5, after LDL (fractions F1–F2) and before albumin (fraction F7).

The technical repeatability of the method was assessed by isolation of lipoproteins from a single plasma pool on 8 separate days over the course of multiple weeks (Fig. 3c). The mean ± standard deviation peak elution volume for the LDL peak, HDL peak, and albumin peak are 8.28 ± 0.0071, 11.7 ± 0.027, and 13.6 ± 0.029 mL, respectively. The % coefficient of variation of these peak elution volumes are 0.09%, 0.23%, and 0.21%, respectively, confirming a high degree of repeatability and column performance.

In order to assess the reproducibility of the method across individual participants who may have different lipid profiles and lipoprotein particle size distributions, plasma samples from 3 healthy individuals from the 10 subjects whose plasma samples were used to generate the plasma pool were isolated using a fractionation approach described in the methods section that was aimed at isolating the LDL, HDL, and albumin peaks at their troughs. (Supplemental Fig. S2, upper panels). SDS-PAGE was run for each fraction from each individual, and showed that the LDL fraction consistently contained ApoB, the HDL fractions consistently contained ApoA-I with no albumin, and the albumin fraction consistently contained albumin (Supplemental Fig. S2).

**Figure 4:**
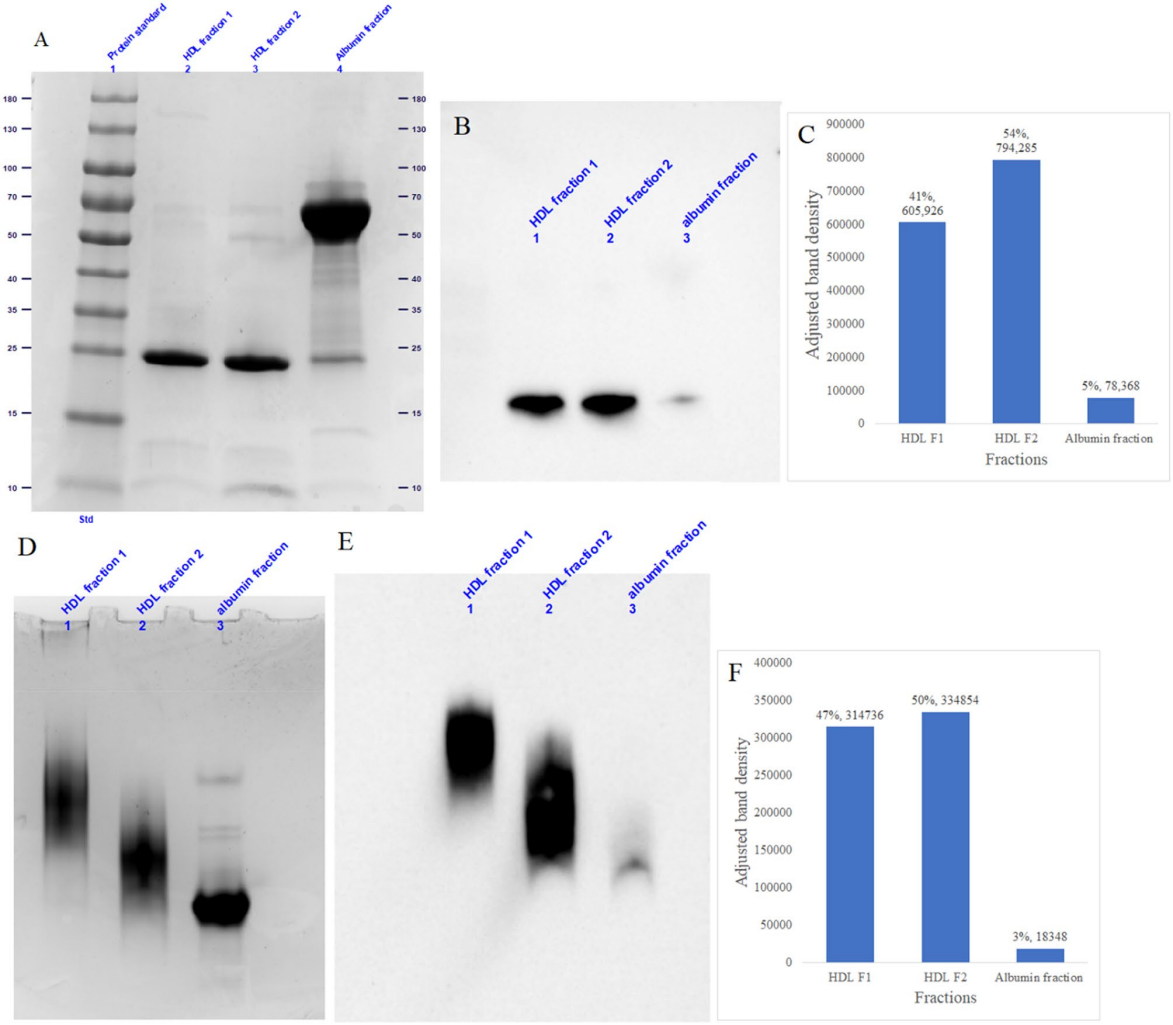
Sodium dodecyl sulfate (SDS)- and native- polyacrylamide gel electrophoresis (PAGE) and Western blot analysis on HDL fraction 1, HDL fraction 2, and albumin fraction. (**A**) SDS-PAGE stained with coomassie blue protein stain. (**B**) Western blot membrane showing ApoA-I proteins transferred from the gel shown in (**A**). (**C**) The relative and absolute value of band density of each ApoA-I band in the Western blot shown in (**B**). (**D**) Native-PAGE stained with coomassie blue protein stain. (**E**) Western blot membrane showing ApoA-I proteins transferred from the gel shown in (**D**). (**F**) The relative and absolute value of band density of each ApoA-I band in the Western blot shown in (**E**).

It is important to note that with this fractionation approach of collecting at the trough between the HDL and albumin peaks, the HDL fractions excluded albumin but there was detectable ApoA-I in the albumin fraction, indicating that some amount of the smallest HDL particles elute in the albumin peak. With this fractionation the elution from 9.78 to 11.52 mL and 11.52 to 13.26 mL was denoted as the HDL1 and HDL2 fractions, and that within 13.26–15.00 mL elution volume was labeled as the albumin fraction. In order to determine the extent of loss of HDL particles into the albumin fraction we performed a Western blot using anti-human ApoA-I antibody on the albumin fraction after denatured- and native-condition PAGE. Figure 4A shows that after denatured PAGE, the albumin fraction contains significant amounts of albumin and much lower amounts of ApoA-I stained by Coomassie blue. The Western blot of this gel shows a minor ApoA-I band in the albumin fraction (lane 3) compared to the ApoA-I bands in the HDL1 and HDL2 fractions (lanes 1 and 2) (Fig. 4B). The densities of the ApoA-I bands in each lane are 41%, 54%, and 5% of the total density respectively for HDL fraction 1, HDL fraction 2, and the albumin fraction (Fig. 4C), demonstrating that about 5% of total ApoA-I was excluded from the HDL1 and HDL2 fractions when the fractionation cutoff was chosen at the trough between the HDL and the albumin peaks. We next determined whether the 5% of ApoA-I that was lost into the albumin fraction from the HDL fractions likely represented free ApoA-I or ApoA-I attached to HDL particles, and if the latter, whether the particles were a subset of particles that was completely different from and unrepresented by those found in the HDL fractions. Using native-condition PAGE we found a range of particles that migrated higher in the gel, consistent with larger HDL particles, as expected in the HDL1 fraction (lane 1), a range of particles that migrated lower in the gel, consistent with smaller HDL particles, as expected in the HDL2 fraction (lane 2), and a major band migrating below the smaller HDL particles in the albumin fraction (lane 3) (Fig. 4D), corresponding to albumin. The Western blot using an ApoA-I antibody on this native gel showed very little signal for ApoA-I in the albumin fraction compared to the two HDL fractions (Fig. 4E). The density of the ApoA-I band in the albumin fraction was 3% of the total ApoA-I signal, compared to 47% in HDL fraction 1 and 50% in HDL fraction 2 (Fig. 4E). Moreover, the ApoA-I stained band in the albumin fraction (lane 3) overlaps with the lowest portion of the band in the HDL2 fraction (lane 2), indicating that the HDL particles present in the albumin fraction are likely to be the smallest HDL particles that are included in the range of particles found in the HDL2 fraction, rather than a separate particle type with a different gel migration pattern as determined by native PAGE. Together, these data confirm that with the isolation method described in this paper using fractionation at the trough between the HDL and albumin peaks there is minimal loss of HDL particles into the albumin fraction, with an estimated 97% of particles being captured in the HDL fractions.

**Figure 5:**
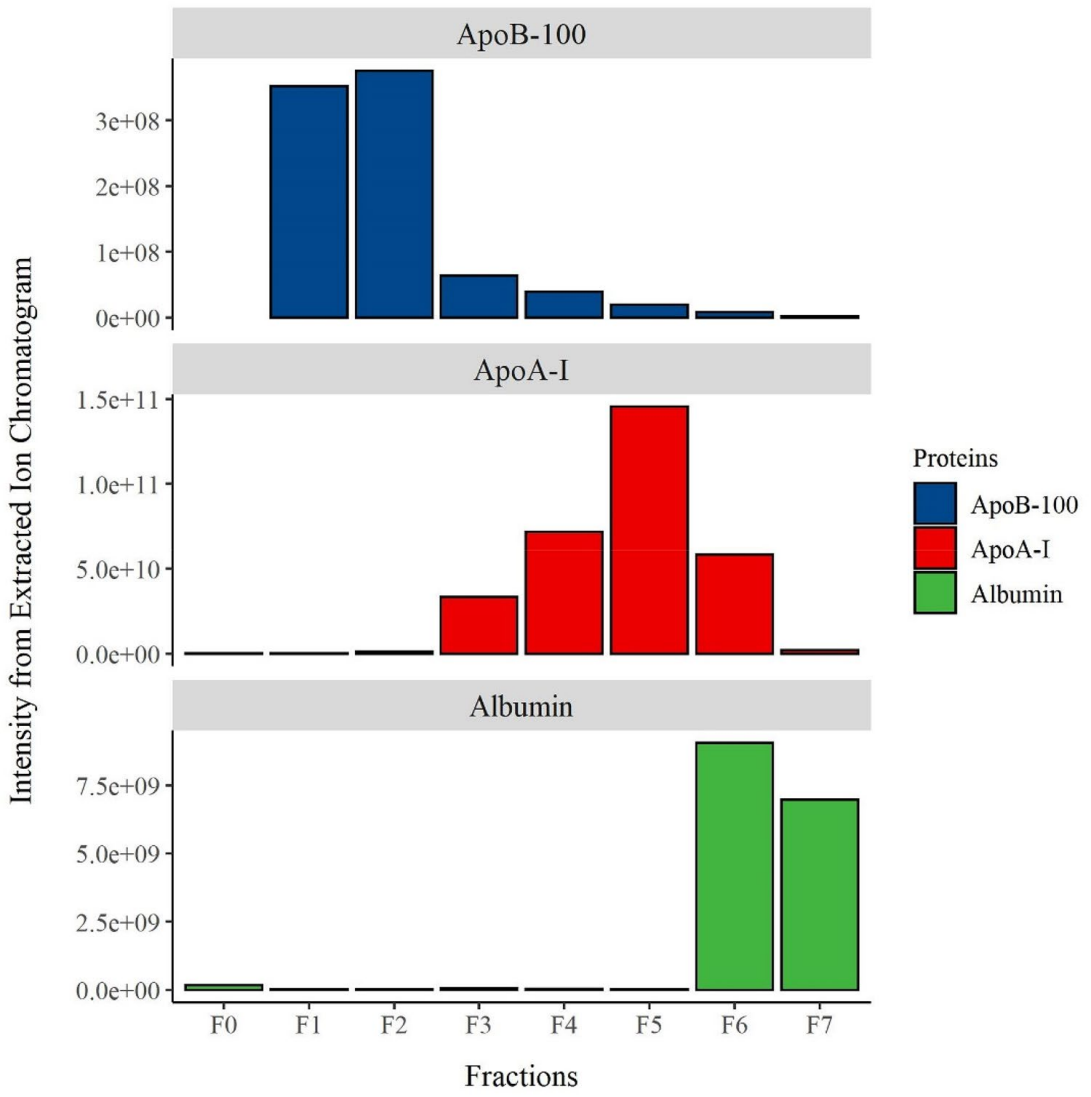
Signal intensity from liquid chromatography-mass spectrometry (LC–MS). Intensity from Extracted Ion Chromatogram (XIC) for (**A**) Apolipoprotein B-100 (ApoB-100), (**B**) ApoA-I, and (**C**) albumin in SEC fractions F0-F7 corresponding to collected low density lipoprotein (LDL), high density lipoprotein (HDL), and albumin (Alb) fractions.

In order to obtain an unbiased assessment of all proteins present in each fraction, proteomics was performed using liquid chromatography-mass spectrometry/mass spectrometry (LC–MS/MS Orbitrap). A total of 48 proteins were detected across all eight SEC fractions (F0-F7, Supplemental Table S2). Figure 5A shows the XIC distributions of ApoA-I, ApoB, and albumin across the SEC fractions. Of the total ApoA-I detected across fractions F0-F7, most of the ApoA-I was found in fractions F3, F4, F5, and F6 respectively which corresponded to the fractions that were expected to contain the HDL particles according to the SEC chromatogram (Fig. 3b) and according to the particle size that is expected to elute in that elution range based on the protein calibration standards (Supplemental Fig. S1). In line with previous literature showing that ApoA-I makes up 60–70% of the total protein in HDL particles^15^, ApoA-I was the most abundant protein constituent in fractions F3, F4, F5, and F6, respectively (Supplemental Table S2). The majority of the protein mass in fraction F6 is albumin, indicating that this smallest fraction of HDL is the most contaminated with albumin. However, as seen in the chromatogram (Fig. 3b), the fractionation that was carried out collecting 1 mL fractions, resulted in the collection of a substantial portion of the albumin peak in fraction F6. Fraction F5 had the highest total intensity for ApoA-I and corresponded to the apex of the HDL peak on the SEC chromatogram. Both F4 and F5 had limited signal for ApoB-100 or albumin, which were several magnitudes lower than that of ApoA-I, indicating that F4 and F5 are the purest and most abundant among the 4 HDL fractions. Only a small proportion of total ApoA-I was detected in fractions F2 (LDL fraction) and F7 (albumin fraction) indicating minimal loss.

ApoB was detected primarily in SEC fractions F1 and F2, corresponding to the LDL peak. ApoB abundance diminished by two orders of magnitude relative to fractions F3-6, the major HDL-containing fractions (Fig. 5B). Albumin was detected in SEC fractions F6, the last HDL fraction which also captured approximately half of the albumin peak, and F7 the fraction containing the albumin peak on the SEC chromatogram. Albumin is nearly undetectable in the rest of the SEC fractions (Fig. 5C).

**Figure 6:**
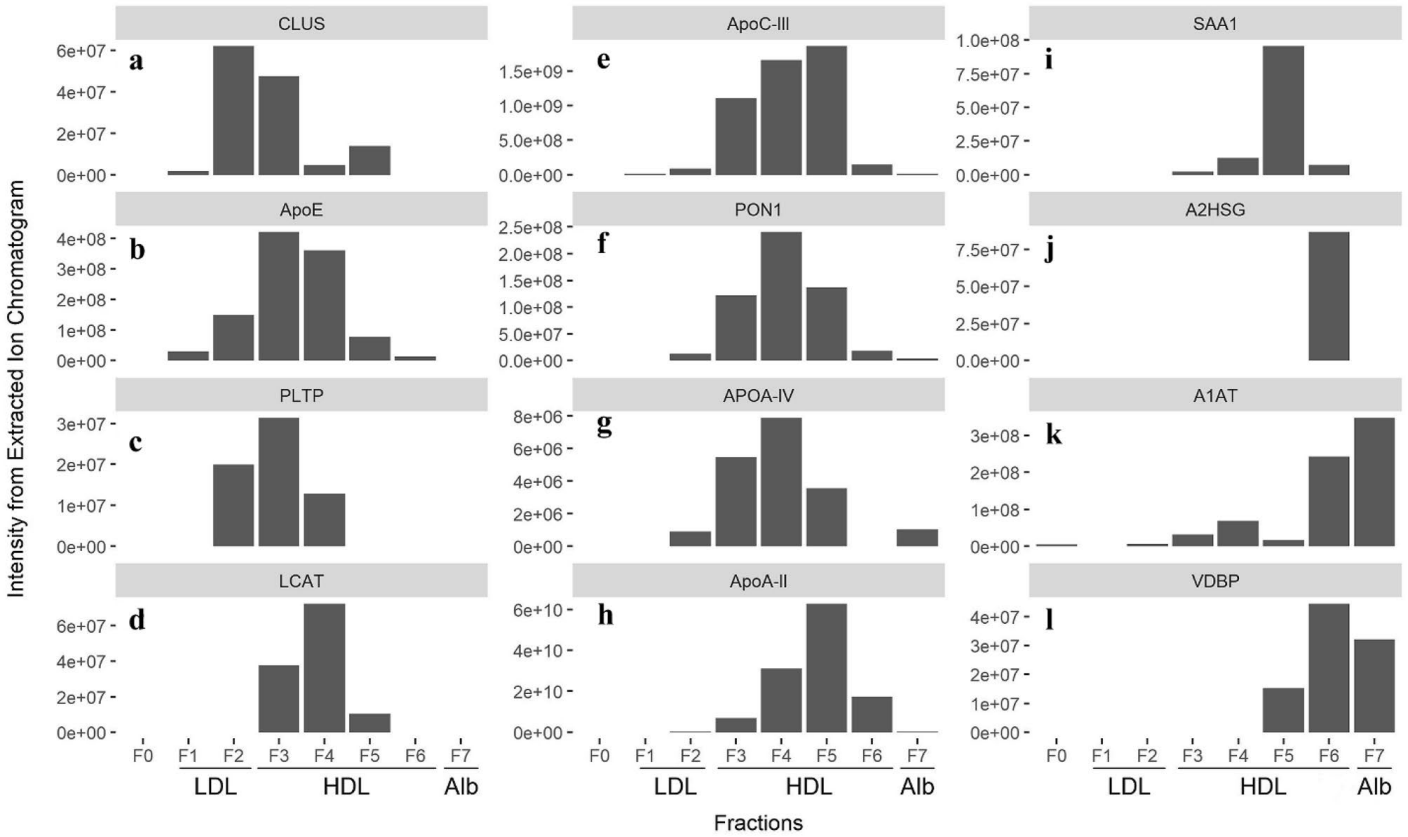
Signal intensity from Extracted Ion Chromatogram (XIC) for 12 selected HDL-associated proteins. (**a**) clusterin (CLUS), (**b**) apolipoprotein E (apoE), (**c**) phospholipid transfer protein (PLTP), (**d**) lecithin cholesterol acyl transferase (LCAT), (**e**) apolipoprotein C-III (apoC-III), (**f**) paraoxonase 1 (PON1), (**g**) apolipoprotein A-IV (ApoA-IV), (**h**) apolipoprotein A-II (ApoA-II), (**i**) serum amyloid A1 (SAA1), (**j**) α-2-HS-glycoprotein (A2HSG), (**k**) α-1-antitrypsin (A1AT), and (**l**) vitamin D binding protein (VDBP). The proteins are displayed such that those enriched in the early fractions corresponding to larger particles are shown in the left column, those distributed across all HDL fractions are shown in the middle column, and those enriched in the later fractions corresponding to smaller particles are shown in the right column. Proteomic analysis for fractions F0–F7 was performed, with fractions F1-F2 corresponding to the low density lipoprotein (LDL) peak, F3-F6 corresponding to the high density lipoprotein (HDL) peak, and fraction F7 corresponding to the albumin (Alb) peak.

In order to determine the compositional heterogeneity of the four HDL fractions, we examined the content of 12 key HDL-associated proteins and their distribution across the SEC fractions, ordered according to their elution time (Fig. 5). Clusterin (CLUS), also known as apolipoprotein J, was found to be enriched in the LDL-containing fraction F2 and the largest HDL fraction F3, with 6 times less found in the later SEC fractions (F4-5) and no detectable levels in the smallest HDL fraction F6 or the albumin fraction F7 (Fig. 6a). Apolipoprotein E (ApoE) was found at highest intensity in fractions F3-4, corresponding with the larger HDL particles, and also LDL fraction F2, with a much smaller intensity in the large LDL fraction F1 (Fig. 6b). Phospholipid transfer protein (PLTP) was enriched in the large HDL fraction F3, followed by the LDL fraction F2, with 3 times less PLTP in HDL fraction F4 and no detectable levels in any of the other fractions (Fig. 6c). Lecithin-cholesterol acyltransferase (LCAT) was found exclusively in HDL fractions F3-5, with the highest intensity found in fraction F4 (large HDL particles), half of the amount was found in fraction F3, and 7 times less found in faction F5 (Fig. 6d). Apolipoprotein C-III (ApoC-III) has a distinct distribution pattern, with an increasing intensity of ApoC-III across the larger HDL fractions F3-5 reaching its peak in fraction F5, and tenfold less ApoC-III in the small HDL fraction F6 and in the LDL fraction F2 (Fig. 6e). Paraoxonase 1 (PON1) was distributed across HDL fractions F3-6 with the highest intensity found in fraction F4, and indicating that PON1 may be enriched in the larger HDL particles (Fig. 6f). Apolipoprotein A-IV (ApoA-IV) was distributed across HDL fractions F3-5, with the highest intensity detected in fractions F3 and especially F4 corresponding to the larger HDL particles, and none found in the small HDL fraction F6 but low intensity found in the albumin fraction F7 (Fig. 6g). ApoA-II was the second most abundant protein, and its distribution across fractions F3-6 indicates its presence across all of the sizes of HDL (Fig. 6h). Serum amyloid A1 (SAA1) was enriched in fraction F5, corresponding to the apex of the HDL peak, with approximately tenfold lower intensity in fraction F4, the larger HDL particles, and about 2 orders of magnitude less SAA1 in fraction F6, the smallest HDL, and no detectable levels found in the LDL or albumin fractions (Fig. 6i). Alpha-2-HS-glycoprotein (A2HSG), or fetuin, was found exclusively in fraction F6, corresponding to the smallest HDL, with no detectable level in any of the other fractions (Fig. 6j). Alpha-1-antitrypsin (A1AT) was found at low intensity in HDL fractions F3-5, peaking in fraction F4, and at about tenfold higher intensity in fractions F6, corresponding to the smallest HDL, and F7, corresponding to albumin, indicating a bimodal distribution for A1AT (Fig. 6k). Vitamin D binding protein (VDBP) on the other hand, was enriched in the smallest HDL fraction F6 and in the albumin fraction F7, with approximately 4 times less found in fraction F5, and no detectable levels in fractions F1-4 (Fig. 6l).

In addition to the XIC data, the full spectral count data generated from the proteomic analysis were included to provide a more comprehensive characterization of the proteins detected in each HDL fraction (Supplemental Table S3). In addition, the spectral count data for each HDL fraction from this study were compared to a few of those reported in previously published studies (Supplemental Table S3). Compared to other published data, the current HDL isolation method yields higher total peptide counts for ApoA-I in all four HDL fractions (F3-6), and ApoA-II in fractions F4 and F5. ApoB total peptide counts are intermediate among the published data selected. Albumin peptide counts are significantly lower in HDL fractions F3, F4, and F5 compared to the majority of previously published data. Likewise, the peptide counts comparison table also shows a significantly lower amount of IgG found in all HDL fractions compared to previously published data.

In order to assess the effectiveness of the SEC step after UC, we carried out an additional SDS-PAGE analysis of the HDL fractions obtained after UC only (Supplemental Fig. S1). The results showed that HDL isolation by UC only without SEC yielded a fraction containing HDL, as indicated by the presence of ApoA-I and ApoA-II (Supplemental Fig. S1). However, there was significant albumin contamination, showing that albumin represented approximately 50% of the total protein content in these fractions. Published UC-only techniques typically contain albumin to ApoA-I ratios of approximately 1:3^3^, whereas our method produced albumin to ApoA-I ratios of 1:50 to 1:195 in fractions F3-F5 respectively.

**Figure 7:**
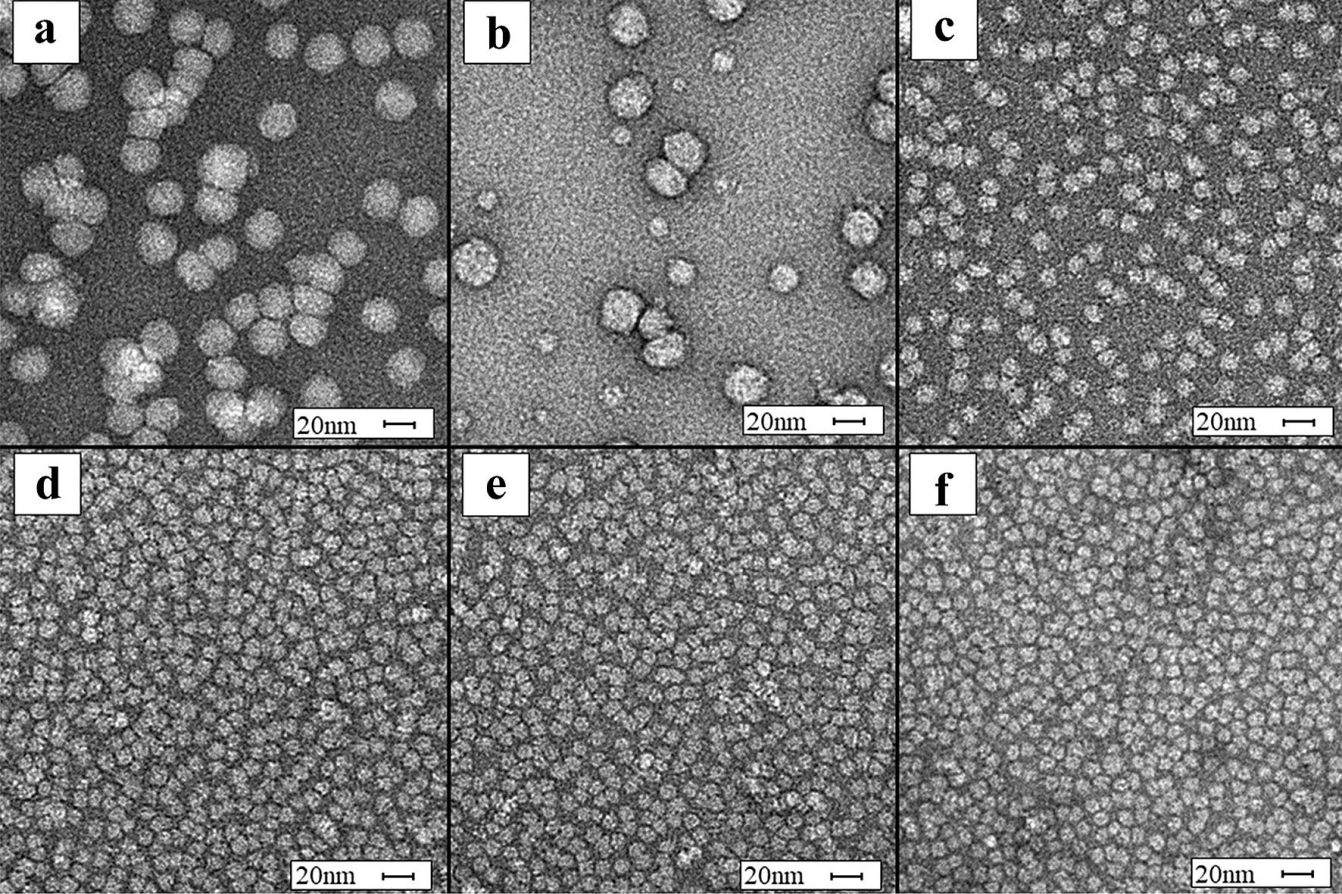
Transmission electron microscopy micrographs of size exclusion chromatography (SEC) fractions. All fractions are displayed with a size bar of 20 nm. (**a**) Fraction F1 shows particles ranging in size from 18 to 28 nm. (**b**) Fraction F2 shows primarily particles in the size range of 18–28 nm, and some particles in the size range of 7–12 nm. (**c**) Fraction F3 shows particles in the size range of 7–18 nm, (**d**) Fraction F4 shows particles in the size range of 7–15 nm, (**e**) Fraction F5 shows particles in the size range of 7–12 nm, and (**f**) Fraction F6 shows particles in the size range of 7–10 nm. Fraction F7 is not displayed because no particles were visible in this fraction.

Some previous HDL isolation methods may disrupt the structural integrity of HDL particles, making them unsuitable for functional analyses where intact particles are needed. We investigated the morphology and size of the isolated particles in the LDL and HDL fractions using TEM. The literature-established size range for HDL was reflected in TEM imaging of the individual fractions (Fig. 7). Fractions F1 and F2 showed particles distinctly larger than F3-F6, approximately 20–30 nm in diameter (Fig. 7a,b), aligning with the literature consensus for LDL particle size^13,19–21^. Fractions F3-F6 (Fig. 7c–f) had distinctly smaller particles than fractions F1 and F2, which fell within the accepted range for HDL diameter, 8–12 nm^13^. Consistent with the size-resolving nature of SEC, the particles visibly diminished in diameter from F3 to F6. Fraction F2 had a mixture of large particles over 20 nm, and small particles under 20 nm, indicating that some HDL-sized particles were co-eluting in the LDL fraction. Importantly, the TEM results indicate that the particles remained structurally intact after the isolation procedure, with a lack of contaminating aggregates and large particles in the HDL fractions.

**Figure 8:**
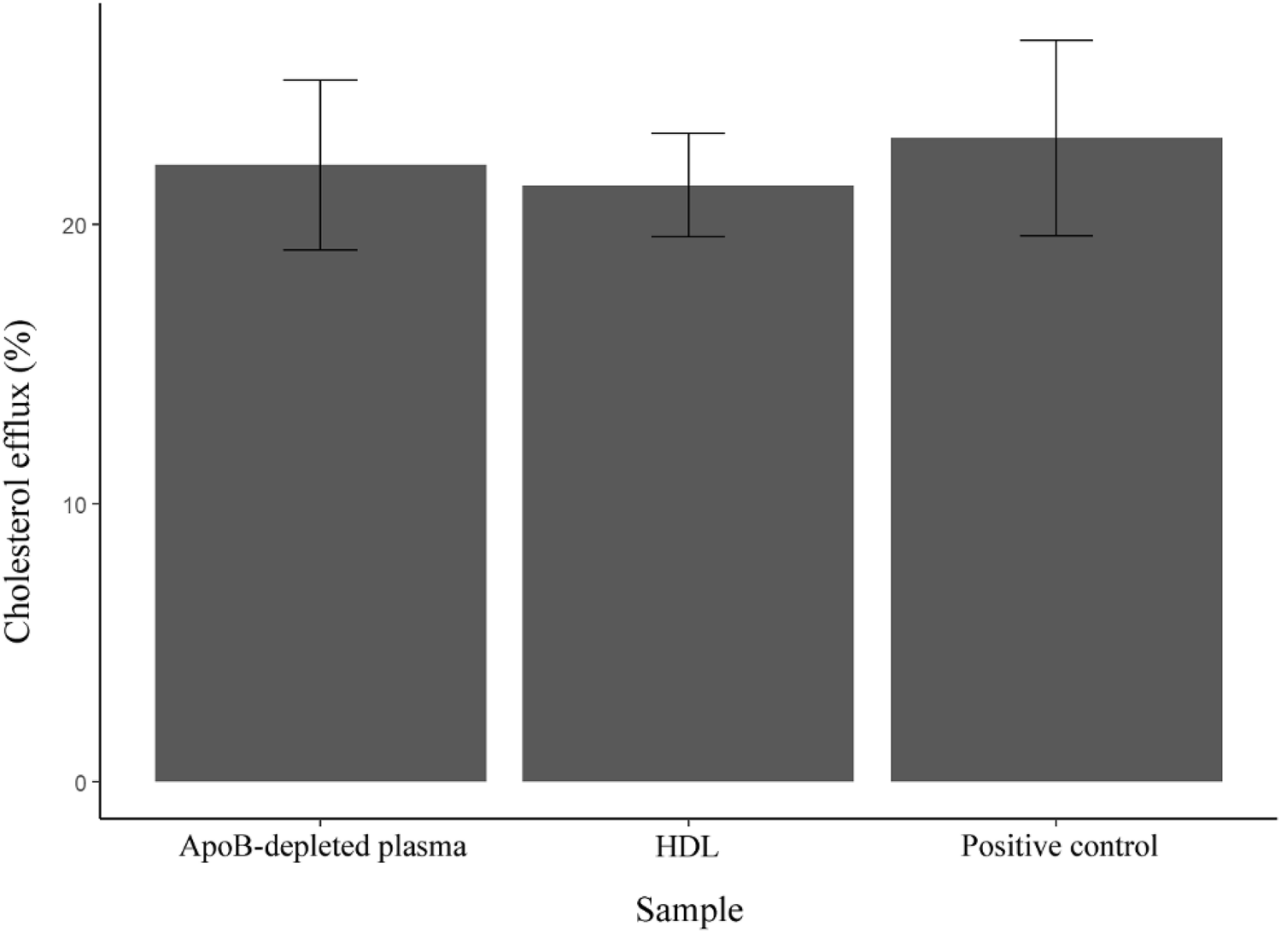
Comparison of the cholesterol efflux capacity of isolated HDL, ApoB-depleted plasma, and a positive control. HDL samples isolated by the current method has a comparable cholesterol efflux capacity (21.4 ± 1.3%) compared to that of ApoB-depleted plasma (22.1 ± 2.5%) or a positive control (23.1 ± 2.9%).

In order to determine the suitability of the method for producing functionally intact particles the cholesterol efflux capacity of the isolated HDL particles was measured and compared to the cholesterol efflux capacity of the ApoB-depleted fraction from the same starting plasma. The cholesterol efflux capacity of the isolated HDL particles was comparable to that of ApoB-depleted plasma at the same protein concentration level (Fig. 8), indicating that the HDL isolated by this method are functionally intact when tested in a cellular assay.

## Discussion

With this HDL isolation method we endeavored to optimize 4 critical objectives simultaneously: (1) Obtain highly purified fractions of HDL that are free of both contaminating larger particles (e.g. LDL) and contaminating plasma proteins (e.g. albumin, immunoglobulin G, other proteins); (2) obtain structurally and compositionally intact particles that are free of salts, chemicals, antibodies, lipid binding agents, and other contaminants that are artifacts of the particle isolation process, so that they are amenable for a variety of downstream compositional and functional analyses; (3) obtain a high yield of HDL particles so that multiple compositional and/or functional analyses can be performed on the same sample and (4) achieve the first 3 objectives with a method that requires low starting plasma volume of 500 µL and that simultaneously preserves the other fractions that contain biologically significant particles. The 500 µL starting plasma volume is optimal in terms of HDL yield for the purpose of collecting a single HDL isolate that can be split into multiple aliquots for multiple compositional, structural, and functional analyses, yet is an acceptable starting volume in most situations including for studies using precious samples from biorepositories and large cohort studies. For example, depending on the plasma sample, on average 400 µg of total protein in the HDL fractions can be obtained from 500 µL plasma sample, and this will provide adequate HDL for several down-stream analyses including for example, cholesterol efflux assay (50 µg), SDS-PAGE/Western blot (10 µg), transmission/cryo-electron microscopy (1–10 µg), proteomics analysis (50 µg) and more. However, in situations where the starting plasma volume needs to be less than 500 µL, plasma can be simply diluted with d = 1.006 g/mL density solution and similar separation can be achieved, albeit with reduced HDL yield.

The results demonstrate minimal contamination by albumin, other plasma proteins, or by ApoB in the HDL fractions F3-F5, with albumin elution in fraction F6 due to this fraction containing a substantial portion of the albumin peak, when using the 1-mL fractionation. However, when using the fractionation approach that collects fractions at the peak troughs, there is no detectable albumin in the HDL fractions (Fig. 4). Failure to deplete plasma proteins, especially albumin, from HDL isolates is a pervasive problem seen in common HDL isolation methods. For example, with the ApoB depletion methods, which utilize polyethylene glycol (PEG), dextran sulfate, or MgCl_2_^22^, although ApoB-containing lipoproteins are effectively depleted, significant amounts of plasma proteins including albumin and immunoglobulins remain^23^. The ApoB-depletion method has the advantages of being fast, easy, and cheap to perform, and may be adequate for quantifying cholesterol and lipidomic analysis of HDL, since the contaminants may not pose significant interference on the analytical results, as the plasma proteins carry little, if any, lipids. However, proteomics analysis and biological functional analysis of HDL require HDL samples high in purity and devoid of functional plasma proteins that can potentially confound the results.

Proteomics was used as an unbiased approach to determine the presence of expected proteins, absence of contaminating proteins, and to estimate the relative abundance of proteins within each of the isolated fractions. The main proteins associated with each particle class, Apo-B for LDL, ApoA-I for HDL, and albumin were enriched in the expected fractions corresponding to the expected elution time based on the SEC protein calibration standards (Supplemental Fig. S3; Fig. 5).

Some HDL-associated proteins were enriched in fractions F3-4 corresponding with the larger HDL particles (Fig. 6a–d), and either depleted in or completely absent from fractions F5-6, indicating that these proteins tend to associate with large HDL particles. For example, CLUS has been found to associate bimodally with large HDL and medium-to-large HDL, but not with the smallest HDL^24^. Our results also showed a bimodal distribution for CLUS (Fig. 6a), with the most eluting in the LDL fraction F2 and the large HDL fraction F3, with an additional though much smaller peak in the medium HDL fraction F5. HDL particles that contain CLUS have been shown to also be enriched in ApoE, ApoC-III, and PON1^10^. We similarly found that ApoE was enriched in the larger HDL fractions. While ApoA-I is distributed in a Gaussian shape and enriched in the middle fractions, corresponding with the abundance of total HDL, ApoE is enriched in the earlier elution fractions F3-4, corresponding with the larger HDL particles (Fig. 6b). Previous studies also showed that ApoE was associated with large HDL particles^25^. A recent study utilizing AF4 to separate 40 individual fractions in 1–2 nm intervals spanning the size range of 7–30 nm followed by LC–MS-based apoprotein quantification, showed that ApoE elutes across this size range, spanning HDL particles, LDL particles, as well as a distinct peak around 15 nm in size (spanning the range of 13–18 nm) where there are low concentrations of ApoA-I, highlighting the presence of ApoE on large HDL particles that are either depleted in or completely free of ApoA-I^13^.

Along with CLUS and ApoE, PLTP was also enriched in the large HDL fraction F3 (Fig. 6c). PLTP facilitates the transfer of phospholipids from lipid-rich lipoproteins to HDL and thereby provides material necessary to expand HDL particle size. LCAT is another enzyme that was found to be enriched in the larger HDL fractions F3 and F4 (Fig. 6d). LCAT is involved in HDL maturation through its function of esterifying cholesterol with a fatty acid from the *sn-*2 position of a phosphatidylcholine. LCAT was absent in many proteomic analyses of HDL^25–27^, which may be due to loss of LCAT using other isolation methods.

ApoC-III is the most abundant ApoC associated with lipoproteins. The main known function of ApoC-III is to inhibit the activity of lipoprotein lipase, delaying the clearance of triglyceride-rich lipoproteins (VLDL and chylomicrons) and has been associated with increased risk of hypertriglyceridemia and poor CVD outcomes^28,29^. ApoC-III-containing HDL were found to be poorly lipidated and to be associated with smaller HDL particles with a density close to 1.21 g/mL^30^. However, a more recent report using AF4-based HDL isolation found ApoC-III to be present across the entire HDL particle size range, with the highest number of ApoC-III molecules per particle found in large HDL particles around 16 nm in size^13^. Our results also indicate the presence of ApoC-III across the HDL size range (Fig. 6e).

PON1 is an enzyme with organophosphatase, oxidase, and phospholipase-like activities that has been associated with the cardio-protective anti-oxidant function of HDL^30^. Studies have suggested that the highly hydrophobic N-terminal of PON1 anchors PON1 onto HDL^31^ but that connection is weak and can be dissociated after prolonged ultracentrifugation times (as long as 48 h)^32^. PON1’s presence in all of the HDL fractions F3-F6 indicates that our separation method is gentle and protective against undesirable loss of HDL-associated proteins. This advantage is important for samples that are separated for further downstream biological analysis.

ApoA-IV is an HDL-associated glycoprotein that is expressed exclusively in the small intestine in humans^33,34^ in response to the ingestion of long-chain fatty acid. A recent study utilizing in situ perfusion of small intestine showed that HDL particles synthesized in the intestinal tract contain ApoA-IV, are enriched in triglyceride, and are smaller and denser than liver-derived HDL particles^35^. ApoA-IV- HDL have been found to be associated with small HDLs of size 7 nm and 8.7 nm^30^. In our study ApoA-IV was distributed across the HDL size range (Fig. 6g). ApoA-II is the second most abundant apolipoprotein found associated with HDL after ApoA-I. It is also associated with the reverse cholesterol transport ability of HDL^36^, however, its exact role has not been fully elucidated. Our results showed that ApoA-II was detected in similar fractions as ApoA-I (Fig. 6h), corroborating previous observations^25,37^.

Some proteins, including SAA1, A2HSG, A1AT and VDBP, were enriched in the small HDL fractions (Fig. 6 i–l). SAA1 is an acute phase protein secreted by the liver during acute inflammatory events and during chronic inflammation in response to cytokine stimulation, and thus has been associated with HDL’s role in modulating immunity^10^. During an active immune response, SAA1 concentration increases significantly and has been suggested to displace ApoA-I in HDL^38,39^. Some evidence suggests that SAA1 is associated with smaller and denser HDL (HDL-3)^38^, which corresponds with our findings of SAA1 enrichment in HDL fraction F5 (Fig. 6i). A2HSG is highly expressed in hepatocytes and is released to the plasma^40^. In our study A2HSG was found exclusively on small HDL particles in fraction F6 (Fig. 6j), the smallest HDL coeluting with albumin. A1AT is a positive acute-phase plasma protein that is protected against oxidation through its association with HDL^6,26^. Our proteomic analysis shows that A1AT is enriched in the smallest HDL fraction F6 (Fig. 6k).VDBP is a carrier protein for Vitamin D metabolites and has been found associated with HDL fractions in some studies^41^. Most bound vitamin D metabolites are carried by VDBP and a small fraction are carried by albumin^42^. In this study VDBP was found in the smallest HDL fractions F5-6 and in the albumin fraction F7 (Fig. 6l). Due to differences in isolation procedures, some studies suggested that SAA1, A2ASG, and A1AT are not HDL-associated proteins, but free plasma proteins that co-isolate as contaminants with HDL^15^. However, other studies suggest otherwise, including an investigation utilizing a sequential antibody-based sandwich ELISA technique which first isolates ApoA-I-containing HDL particles followed by a second antibody-based capture of A1AT-containing HDL^10^. Using this technique, the authors showed that although most of the A1AT is indeed present as free protein in plasma, a small proportion of it does associate with a subset of HDL particles.

As shown by the differential distribution of specific proteins across the entire size range of HDL, the analysis of functions and characteristics of these different HDL subclasses may be important in gaining a better understanding of the complex biology of HDL. With the method described here, it is possible to fine-tune the size-based fractions of HDL collected. If the objective of the study is to collect the entirety of the HDL pool from a given plasma sample, it is possible to pool fractions F3-F6 corresponding to the HDL peak together. This approach maximizes total HDL amount and representativeness of the array of particles present in the plasma sample, while minimizing contamination compared to comparable methods. Alternatively, it is also possible to adjust the fractionation to collect just the middle of the HDL peak (F4 and F5), which would minimize contaminants (LDL and albumin) but would slightly reduce yield and leave out some of the heterogeneity in the HDL particles. The range of HDL fractions can be further adjusted deliberately to meet the investigators’ specific needs. We showed an example of setting the fractionation cutoff at the troughs between the LDL and HDL peaks, and between the HDL and albumin peaks, and found little evidence of albumin contamination in the HDL fractions, while also minimizing loss of HDL in the albumin fraction (Fig. 4). Whether contaminating albumin is a concern depends on the purpose of the experiment. Compositional studies, such as proteomic and glycomic analysis of HDL may not require albumin to be eliminated from the sample since albumin fragments generated from sample preparation and ionization during MS analysis can be detected and eliminated manually or by setting specific parameters in the software programs. On the other hand, for functional assays, especially if the approach is to apply a consistent dose of HDL as measured by total protein content, differential contamination levels by albumin can impact the relative proportion of HDL particles loaded per well.

Because ApoA-I was detected both by SDS-PAGE and shotgun proteomics in the albumin fraction, we set out to determine the extent of loss of HDL particles using native-condition PAGE and Western blot with anti-ApoA-I antibody. We showed that with our isolation method, using a fractionation approach where the HDL fraction is collected at the trough between the HDL and albumin peaks, there is minimal loss of HDL (estimated at 3%) into the albumin fraction (Fig. 4).

There are some limitations and further considerations to take into account with the current method. One of the pitfalls of ultracentrifugation is the potential loss of HDL protein components, which under conditions of high salt and high g forces, may disassociate from the HDL particle^17,43^. The current method minimizes the duration of ultracentrifugation in the first step to a total of 4 h, compared to as long as 48 h required in other methods^5^. Although some HDL-associated protein loss may still occur, we have demonstrated that our method does not lead to the complete loss of functionally significant HDL-associated proteins, including PLTP, PON1, and LCAT. We have observed that the SEC chromatogram and isolation efficiency is affected by the lipid profile of the plasma sample. A plasma sample from an overnight fasted healthy subject is likely to produce a chromatogram similar to the pooled plasma analyzed in this study; however, a plasma sample from a hyperlipidemic or dyslipidemic subject may result in a chromatogram that is significantly different in peak shape, yield, and sometimes the ideal retention time cutoffs compared to plasma from fasted, healthy subjects. Thus, adjustment of fraction collection geared toward the sample type to be run may be necessary for sample sets including plasma samples from individuals with different types of hyperlipidemia, dyslipidemia, and/or postprandial samples. XIC data from untargeted proteomic analysis, although arguably more quantitative than spectral counts^15^, are not quantitative due to inherent differences in the ionizatin efficiencies of different peptides, which are unrelated to their abundance. Therefore, untargeted proteomics was not used in this study to measure the absolute quantities of the 48 proteins that were detected in the fractions, but to estimate their relative abundance and relative distribution across the fractions. Finally, although our method depletes plasma proteins such as IgG from the HDL fractions comparably or more robustly compared to other published methods, there were still small amounts of IgG detectable by proteomics (Supplemental Tables S2 and S3).

## Supplementary Information

Supplementary Figures.

Supplementary Table 1.

Supplementary Table 2.

Supplementary Table 3.

## Data Availability

All data are available as supplemental files.
