# Supplementary material for: Isolation of HDL by sequential flotation ultracentrifugation followed by size exclusion chromatography reveals size-based enrichment of HDL-associated proteins
Source: Sci Rep. 2021 Aug 9;11:16086. doi: 10.1038/s41598-021-95451-3 (PMC8352908; doi:10.1038/s41598-021-95451-3)
Supplement: Supplementary file 1 — Supplementary Figures. [file 41598_2021_95451_MOESM1_ESM.docx]

**An optimized HDL isolation method utilizing sequential flotation density ultracentrifugation and size exclusion chromatography reveals size-based enrichment of HDL-associated proteins**

Short title: An optimized method to isolate high-density lipoprotein from plasma

Jack Jingyuan Zheng^a^, Joanne K. Agus^a^, Brian V. Hong^a^, Xinyu Tang^a^, Christopher H. Rhodes^a^, Hannah E. Houts^a^, Chenghao Zhu^a^, Jea Woo Kang^a^, Maurice Wong^b^, Yixuan Xie^b^, Carlito B. Lebrilla^b^, Emily Mallick^c^, Kenneth W. Witwer^c^, Angela M. Zivkovic^a*^


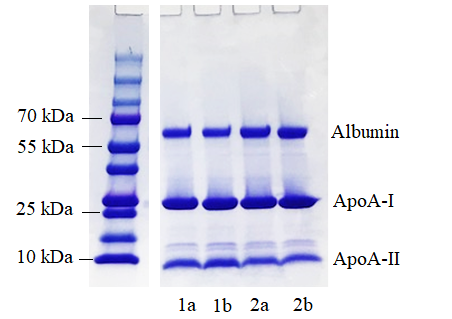


**Supplemental Figure 1: SDS-PAGE image for HDL fractions isolated by sequential floatation ultracentrifugation only.** Lanes labeled 1a, 1b, 2a, and 2b were from two different subjects. The gel image shows clear bands for albumin (around 65 kDa)), ApoA-I (around 28 kDa)), and ApoA-II (around 11 kDa).


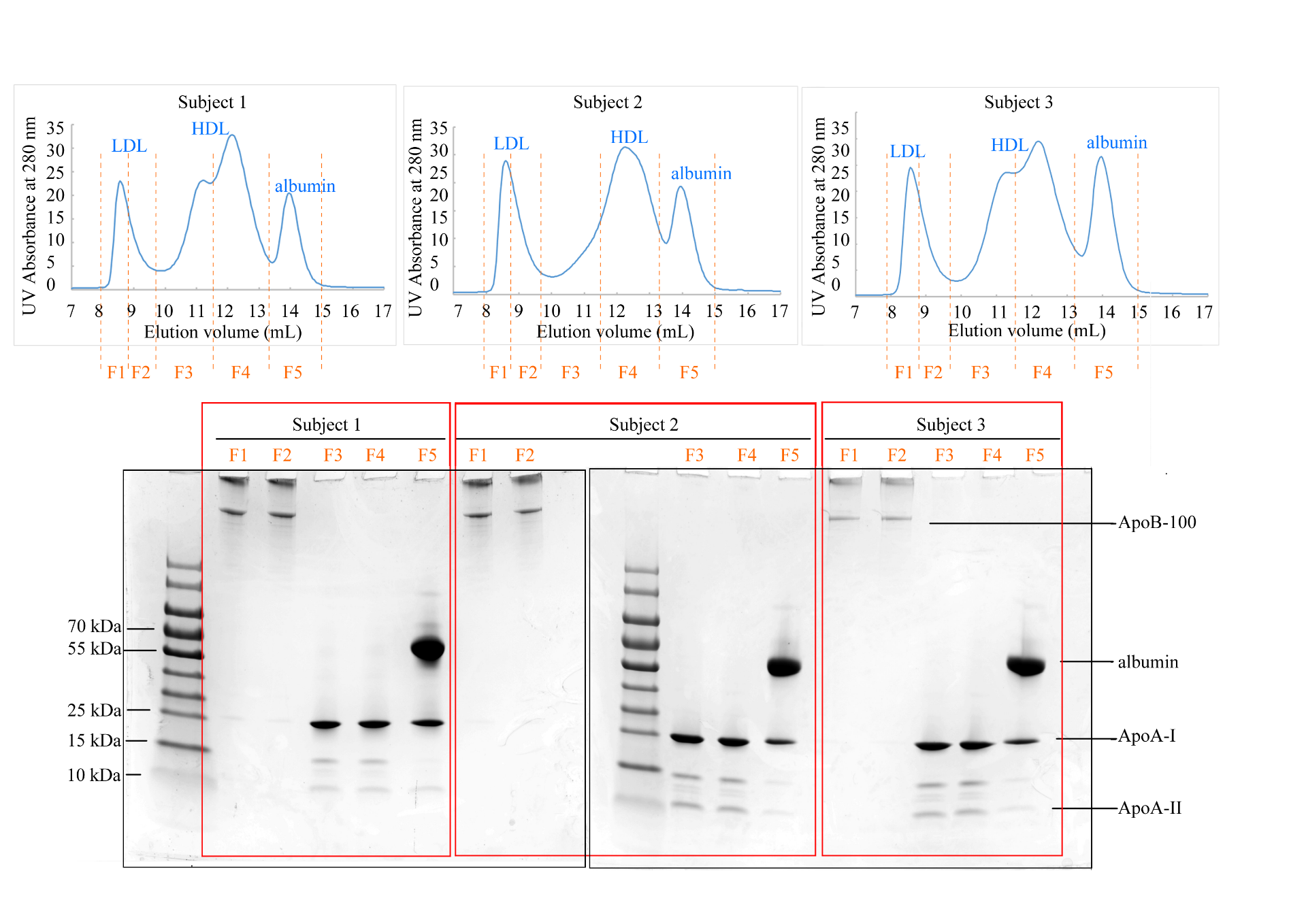


**Supplemental Figure 2: SEC chromatograms and SDS-PAGE images for the isolated LDL fractions, HDL fractions, and albumin fraction from plasma samples from three healthy subjects.** Fractionation was slightly adjusted to include two LDL fractions, 7.95-8.85 mL (F1) and 8.85-9.78 mL (F2), two HDL fractions, 9.78 – 11.52 mL (F3) and 11.52 – 13.26 mL (F4), and one albumin fraction, 13.26 – 15.00 mL (F5). Two separate SDS-PAGE gel images are shown in the lower panel. The first gel included Fraction 1-5 from subject 1 and Fraction 1-2 from subject 2. The second gel included Fraction 3-5 from subject 2 and Fraction 1-5 from subject 3. The lanes corresponding to each subject is indicated by the red boxes, and each individual gel is indicated by the black boxes. SDS-PAGE separates the isolates into clear bands of ApoB for LDL fractions, ApoA-I and ApoA-II for HDL fractions, and albumin and ApoA-I in albumin fractions.

**
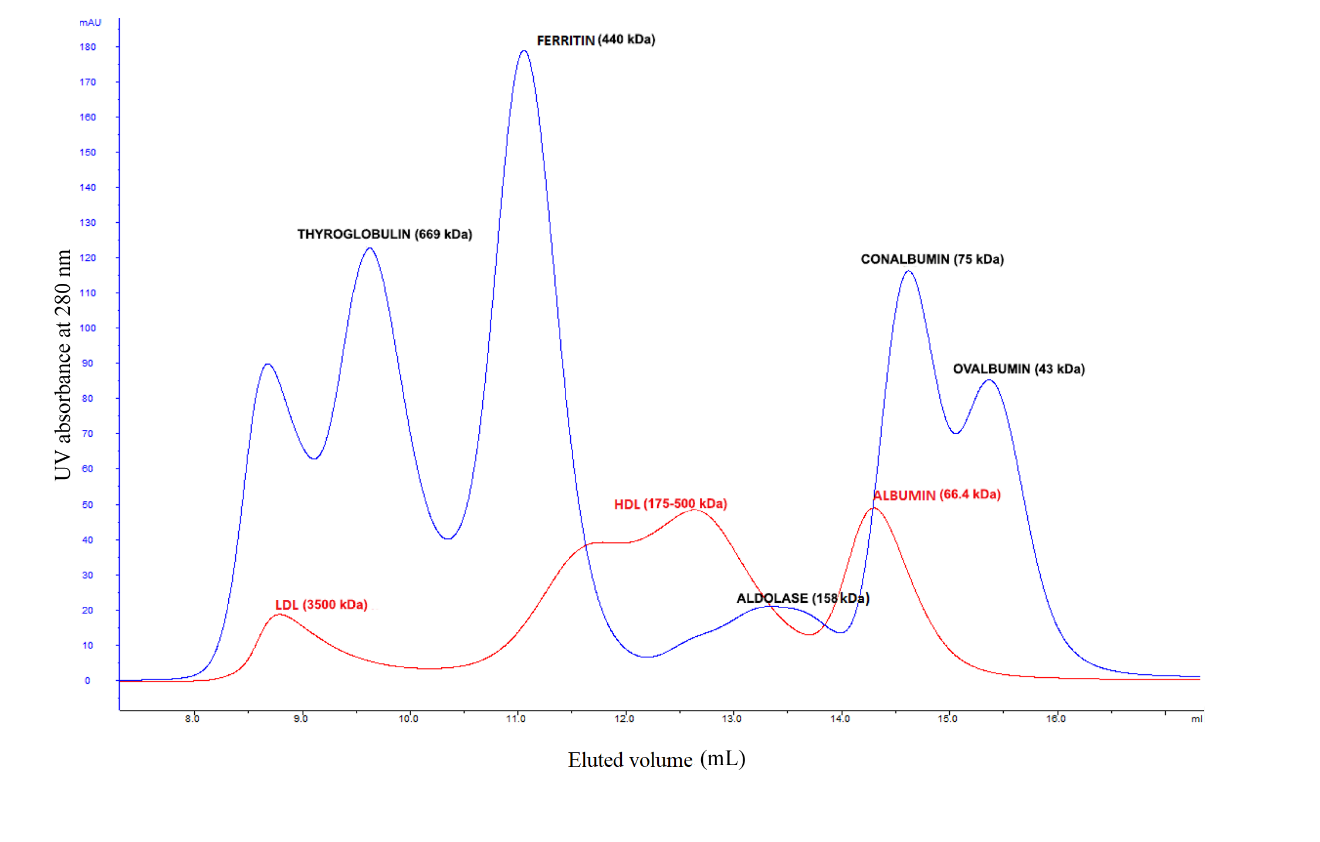
**

**Supplemental figure 3. Particle size from the fractionated eluent compared with a protein molecular weight standard on a UV chromatogram (280 nm).** An FPLC run using a mixture of protein molecular weight standard (thyroglobulin at 669 kDa, ferritin at 440 kDa, aldolase at 158 kDa, conalbumin at 75 kDa, and ovalbumin at 43 kDa) confirmed that the particles in the respective fractions have the correct molecular weight (LDL at 3,500 kDa, HDL at 175 – 500 kDa, and albumin at 66.4 kDa).
